# Serial Changes of Neointimal Tissue after Everolimus-Eluting Stent Implantation in Porcine Coronary Artery: An Optical Coherence Tomography Analysis

**DOI:** 10.1155/2014/851676

**Published:** 2014-09-18

**Authors:** Hoyoun Won, Jung-Sun Kim, Dong-Ho Shin, Byeong-Keuk Kim, Young-Guk Ko, Donghoon Choi, Yangsoo Jang, Myeong-Ki Hong

**Affiliations:** ^1^Chung-Ang University Medical Center, 102 Heukseokro, Dongjak-gu, Seoul 156-755, Republic of Korea; ^2^Division of Cardiology, Department of Internal Medicine, Severance Cardiovascular Hospital, Yonsei University College of Medicine, Yonsei University Health System, 250 Seongsanno, Seodaemun-go, Seoul 120-752, Republic of Korea; ^3^Cardiovascular Institute, Yonsei University College of Medicine, 250 Seongsanno, Seodaemun-go, Seoul 120-752, Republic of Korea; ^4^Severance Biomedical Science Institute, Yonsei University College of Medicine, 250 Seongsanno, Seodaemun-go, Seoul 120-752, Republic of Korea

## Abstract

*Purposes*. The serial changes in neointimal tissues were compared between everolimus-eluting stent (EES) and bare-metal stent (BMS) in the porcine coronary artery using optical coherence tomography (OCT). *Methods*. Serial (1, 3, and 6 month follow-up after stent implantation) OCT examinations were performed in 15 swine with 15 BMS- and 15 EES-treated lesions in porcine coronary arteries. *Results*. In BMS-implanted lesions, neointimal volume decreased from 7.3 mm^3^ to 6.9 mm^3^ and 6.4 mm^3^ at 1, 3, and 6 months follow-up without statistical significance (*P* = 0.369). At the time points of 1, 3, and 6 months, neointimal tissue appearance was mainly a homogeneous pattern (80.0%, 93.3%, and 100%, resp.), while the other pattern was layered. In contrast, in EES-implanted lesions, neointimal volume significantly increased from 4.8 mm^3^ to 9.8 mm^3^ between 1 and 3 months but significantly decreased to 8.6 mm^3^ between 3 and 6 months (*P* < 0.001). Between 1 and 3 months, the layered pattern of neointimal tissue increased from 26.7% to 66.7% but decreased to 20.0% between 3 and 6 months. *Conclusions*. EES had a biphasic pattern of neointimal amounts that correlated with changes in neointimal morphology.

## 1. Introduction

The development of drug-eluting stents (DES) has significantly reduced the incidence of in-stent restenosis compared to bare-metal stents (BMS) [1, 2]. However, even in the DES era, in-stent restenosis remains a significant clinical problem, particularly in high-risk patients [3]. Optical coherence tomography (OCT) is a high-resolution imaging tool that is ideal for the evaluation of neointimal tissue characteristics as well as strut coverage of coronary stents [4–6]. The everolimus-eluting stent (EES) is a second-generation DES with a target lesion revascularization rate as low as 4.7% during the 5-year clinical follow-up [7]. However, there are few studies evaluating temporal changes in neointimal tissue after EES implantation. The purpose of this study was to evaluate serial changes in neointimal tissue in stented segments based on OCT between 1, 3, and 6 months after EES implantation compared to BMS implantation in normal porcine coronary arteries.

## 2. Materials and Methods

### 2.1. Study Design

A total of 15 swine (weighing 25 to 30 kg) were studied for 6 months to evaluate neointimal tissue serially following EES implantation. Two stent types were implanted: EES (Xience Prime, 3.0 × 12 mm, Abbott Vascular, Santa Clara, CA) as a target stent and BMS (Blazer, 3.0 × 13 mm, OrbusNeich, Hong Kong) as a control stent. Each stent was deployed in the left anterior descending artery or right coronary artery. At the time of stent deployment, each stent was systematically randomized to each coronary artery in each animal. Follow-up angiography with OCT was serially performed 1, 3, and 6 months after stent implantation. All animals received humane care in compliance with the Animal Welfare Act and “The Guide for the Care and Use of Laboratory Animals” formulated by the Institute of Laboratory Animal Research [8]. This study was approved by the local institutional animal care and use committee (Medi Kinetics, MK-IACUC: 111027-0001 and Cardiovascular Production Evaluation Center, Yonsei University College of Medicine).

### 2.2. Procedural Description

All animals were premedicated at least 12 hours before the procedure with 100 mg aspirin and 300 mg clopidogrel. Anesthesia was performed via intramuscular injection of ketamine (20 mg/kg) and xylazine (2 mg/kg). After adequate anesthesia, animals were intubated and inhaled isoflurane (1-2%) delivered through a precision vaporizer and a circle absorption breathing system with periodic arterial blood gas monitoring. Under sterile conditions, arteriotomy of the carotid artery was performed and a 6-Fr vascular access sheath was introduced into the carotid artery. Vital signs were continuously monitored using surface electrocardiography and were recorded at approximately 20 min intervals. Unfractionated heparin (5,000 to 10,000 IU) was administered to maintain activated clotting times of 250 to 300 seconds. Using fluoroscopic guidance, stent implantation on coronary arteries was performed with a stent-to-artery ratio of 110% to 120% overstretch for full apposition at predetermined sites using conventional techniques. After stent implantation, carotid arteries were repaired and the incision site was closed with adequate suture material until the next use of the carotid arteries. All animals received 100 mg aspirin and 75 mg clopidogrel daily after stent implantation. They were fed a regular diet throughout the duration of the study [4].

### 2.3. OCT Procedure and Analyses

OCT was performed using the C7-XR imaging systems (LightLab Imaging, Inc., St. Jude Medical, St. Paul, MN). The OCT catheter was pulled back at 20 mm/s and OCT images were generated at 100 frames/s. Contrast media were continuously flushed through a guiding catheter at a rate of 4 to 5 mL/s for 3 to 4 seconds. Images were continuously acquired and stored digitally for subsequent analyses. All OCT images were analyzed at a core laboratory (Cardiovascular Research Center, Seoul, Republic of Korea) by analysts who were blinded to procedural information. Cross-sectional OCT images were analyzed at 1 mm intervals. Stent and luminal cross-sectional areas (CSA) were measured and neointimal hyperplasia (NIH) CSA was calculated by subtracting the luminal CSA from the stent CSA. The NIH CSA percentage was calculated as the NIH CSA divided by the stent CSA. NIH thickness was measured as the distance between the strut with a line perpendicular to the neointima and the endoluminal surface of the neointima. The NIH volume was calculated as NIH CSA × length. The maximal NIH site with 2 mm proximal and distal adjacent portion in the stented segment was serially compared to the corresponding segment based on the maximal NIH site at the 1 month follow-up. Serial comparisons were performed by matching the length from the stent edge and anatomical features, such as side branch.

**Figure 1:**
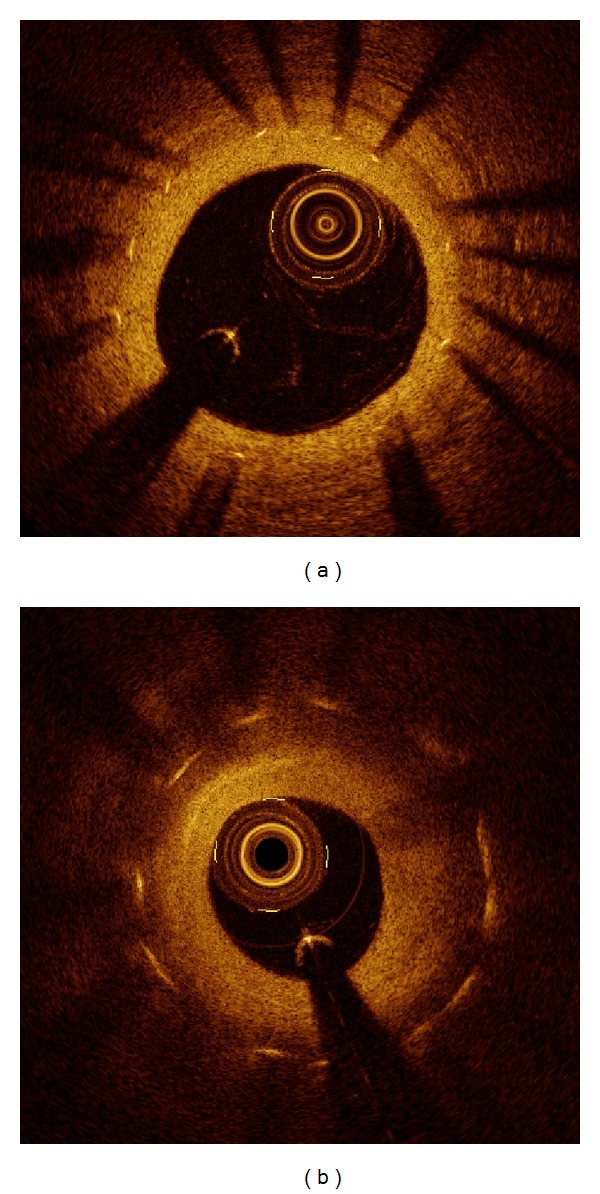
Representative optical coherence tomographic images of neointima. Homogeneous pattern (a) and layered pattern (b).

If the mean neointimal thickness was ≥20 *μ*m, neointimal tissue characteristics were qualitatively classified as homogeneous, heterogeneous, or layered pattern. Homogeneous patterns were defined as uniform optical neointima tissue properties without focal variation in backscattering pattern. Heterogeneous patterns were defined as focal changes in optical properties and various backscattering patterns. Layered pattern refers to concentric layers with different optical properties, namely, an adluminal high scattering layer and abluminal low scattering layer (Figure 1) [9].

### 2.4. Quantitative Angiographic Analyses

Quantitative coronary angiography analyses were performed using an offline computerized quantitative coronary angiographic system (CASS System, Pie Medical Imaging, Maastricht, The Netherlands) in an independent core laboratory (Cardiovascular Research Center, Seoul, Korea). The minimal lumen diameter and reference diameters of treated coronary lesions were measured in the view with the narrowest lumen and the least amount of foreshortening.

### 2.5. Statistical Analyses

Continuous variables are expressed as a median with interquartile range (IQR). Wilcoxon signed-rank tests or Friedman tests were used to compare variables in a longitudinal manner. Categorical variables are expressed as both numbers and percentages and were compared using Fisher's exact tests. Data were analyzed using SPSS 18.0 software for Windows (SPSS, Chicago, IL). A *P* value < 0.05 was considered statistically significant.

## 3. Results and Discussion

### 3.1. Results

**Figure 2:**
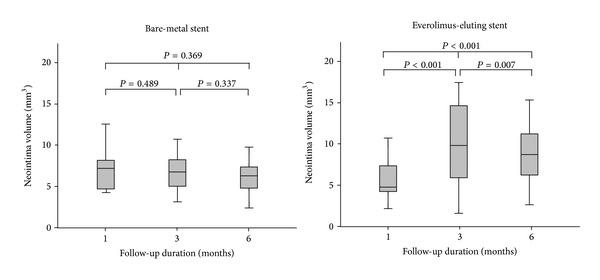
Serial changes in neointimal volume at the segment with maximal amounts of neointimal hyperplasia at the 1-month follow-up.

**Figure 3:**
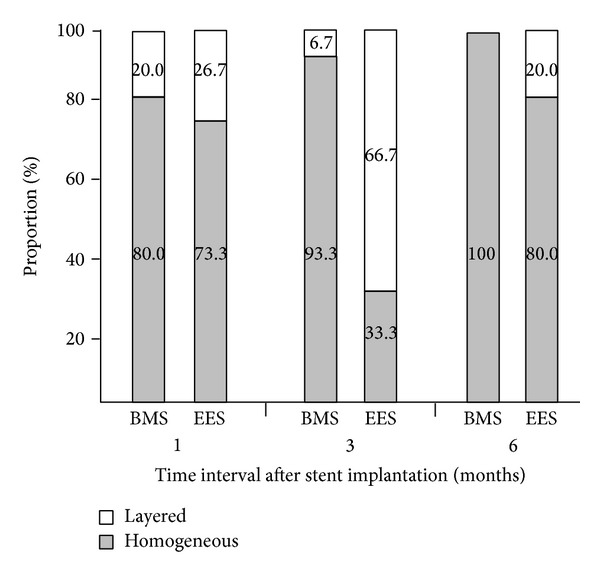
Serial changes in neointimal patterns at the segment with maximal amounts of neointimal hyperplasia at the 1-month follow-up. BMS, bare-metal stent; EES, everolimus-eluting stent.

**Figure 4:**
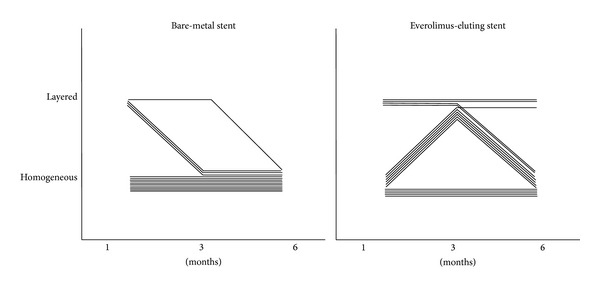
Evolution of serial changes in neointimal patterns in bare-metal stent and everolimus-eluting stent.

**Figure 5:**
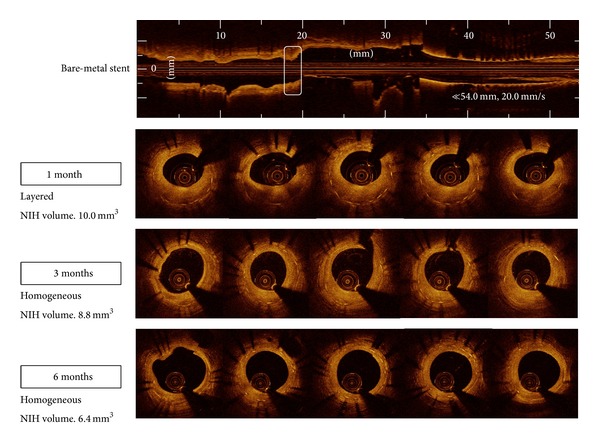
Examples of neointimal volume and patterns at the segment with maximal amounts of neointimal hyperplasia at 1, 3, and 6 months following bare-metal stent implantation. NIH, neointimal hyperplasia.

**Figure 6:**
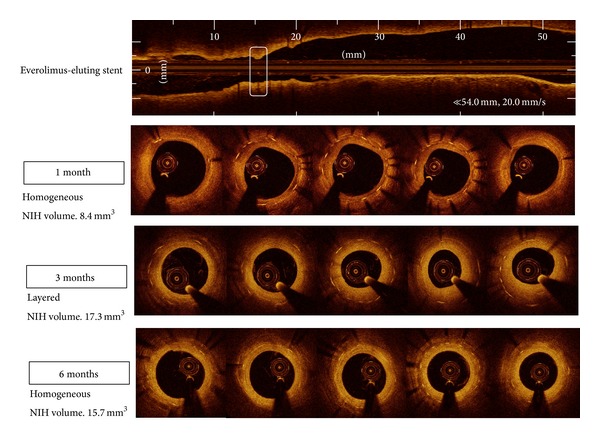
Examples of neointimal volume and patterns at the segment with maximal amounts of neointimal hyperplasia at 1, 3, and 6 months following everolimus-eluting stent implantation. NIH, neointimal hyperplasia.

EES was implanted in eight left anterior descending arteries and seven right coronary arteries, whereas BMS was deployed in seven left anterior descending arteries and eight right coronary arteries. Balloon-to-artery ratio was similar between two groups (1.11 ± 0.06 in BMS versus 1.14 ± 0.04 in EES, *P* = 0.189). Serial OCT evaluation of NIH was assessed in a total of 15 BMS- and 15 EES-implanted into the coronary arteries of 15 animals after 1, 3, and 6 months. Serial angiographic and OCT findings are shown in Table 1. Based on analyses of whole stented segments in BMS, the mean NIH thickness was maximal at 1 month (190.4 *μ*m, IQR 127.0–243.9 *μ*m) and decreased at 3 and 6 months (182.2 *μ*m, IQR 131.9–229.4 *μ*m and 169.8 *μ*m, IQR 112.7–211.1 *μ*m, *P* = 0.189). However, in EES, it was maximal at 3 months (276.5 *μ*m, IQR 172.2–428.1 *μ*m) and decreased at 6 months (269.6 *μ*m, IQR 199.9–396.4 *μ*m, *P* = 0.100). For analyses of maximal NIH segments at 1 month, serial changes in NIH volume after BMS and EES are shown in Figure 2 and Table 2. In BMS-implanted lesions, NIH volume decreased from 7.3 mm^3^ (IQR 4.7–8.4 mm^3^) at the 1-month follow-up to 6.9 mm^3^ (IQR 4.6–8.2 mm^3^) and 6.4 mm^3^ (IQR 4.3–7.4 mm^3^) after 3 and 6 months, respectively (*P* = 0.369). At the different time points of 1, 3, and 6 months, neointimal tissue appearance was predominantly the homogeneous pattern (80.0%, 93.3%, and 100%, resp.), while the other pattern was layered (Figure 3). In contrast, in EES-implanted lesions, NIH volume significantly increased from 4.8 mm^3^ (IQR 4.1–7.6 mm^3^) to 9.8 mm^3^ (IQR 5.7–15.1 mm^3^) between 1 and 3 months (*P* < 0.001) but significantly decreased to 8.6 mm^3^ (IQR 6.0–11.2 mm^3^) between 3 and 6 months (*P* = 0.007) (*P* < 0.001 between 1 and 6 months) (Figure 2). Between 1 and 3 months, the layered pattern of neointima increased from 26.7% to 66.7% but decreased to 20.0% between 3 and 6 months (Figures 3 and 4). Figures 5 and 6 show typical OCT images of serial NIH changes in BMS- and EES-implanted coronary arteries, respectively.

### 3.2. Discussion

Using serial (1, 3, and 6 months follow-up) OCT in the nonatherosclerotic porcine coronary artery model, the present study showed that (1) a biphasic neointimal response of EES consisted of an early progression phase and late regression phase and (2) quantitative neointima changes correlated with morphological neointima changes from a layered pattern to a homogeneous pattern.

Serial quantitative neointimal changes of BMS have been well evaluated in human studies. In BMS, long-term angiographic luminal responses were a triphasic pattern that consisted of a restenosis phase at the early period for 6 months, a regression phase at the intermediate period between 6 months and 3 years, and a late renarrowing phase after 4 years [10]. The results of intravascular ultrasound studies were also consistent with those from a previous angiographic study [11, 12]. One intravascular ultrasound study reported that mean neointimal CSA significantly decreased from 2.6 ± 1.0 to 2.3 ± 0.9 mm^2^ between 6 and 24 months after BMS-implantation [12]. The results from the BMS-implanted lesions in the current study are in agreement with results from previous studies [10–12].

However, information regarding long-term serial changes in neointima following DES implantation was not sufficient. Sousa et al. first reported after 4-year angiographic follow-up of 26 sirolimus-eluting stent-treated patients that a temporal progression of late loss was observed: late loss was 0.08 mm from postintervention to the 1-year follow-up, and it was −0.03 mm from 1- to 2-years and 0.20 mm from 2- to 4-years [13]. In another study with 15 sirolimus-eluting stent-treated patients, there were no significant changes in stent minimal lumen diameter based on quantitative coronary angiographic analyses and no significant deterioration in lumen volume by intravascular ultrasound measurement between the 6-month and 2-year follow-up [14]. In substudy of randomized TAXUS II trial, neointimal volume measured by intravascular ultrasound increased significantly from 10.13 mm^3^ at 6 months to 15.11 mm^3^ at 2 years after paclitaxel-eluting stent implantation (*P* = 0.0011) [15]. In 2-year follow-up results of the SPIRIT II trial, in-stent neointimal volume progressed from 4.13 mm^3^ at 6 months to 8.42 mm^3^ at 2 years after EES implantation [16].

Quantitative OCT assessment has been well validated. One study of* in vivo* OCT comparison with histology in a rabbit carotid model showed that histological and OCT measurements of mean NIH thickness were similar and closely related (*r* = 0.85, *P* < 0.001) [17]. OCT and histology correlated highly for the evaluation of neointimal area (*R* ^2^ = 0.804), luminal area (*R* ^2^ = 0.825), and neointimal thickness (*R* ^2^ = 0.789) in a normal porcine coronary model [4]. In qualitative OCT assessments, neointima with rich smooth muscle cells and dense collagen fibers has a high backscatter signal and is shown as high optical intensity, extracellular matrix or myxomatous tissue, such as proteoglycan or fibrin rich neointima with few smooth muscle cells, and has a low backscatter signal and is shown as a lucent signal [18–20]. Although a homogeneous pattern with a high optical intensity is usually observed in in-stent restenosis of BMS, the layered pattern was observed more frequently in in-stent restenosis of DES [18]. The layered pattern consisted of a high intense inner layer with smooth muscle cells and a low intense outer layer with extracellular material rich lesions [18]. In current study, heterogeneous pattern was not detected in contrast to the previous study [20], which could not be explained exactly. The previous study used hypercholesterolemic swine model and different type of DES (especially, paclitaxel-eluting stent), which was evident difference compared to our study [20].

Using intravascular imaging tools, serial follow-up data beyond 2 years after DES implantation are sparse in clinical practice as well as in random clinical trials due to the practical or ethical aspects of clinical practice. However, these data are very important for understanding the long-term natural history of DES-treated patients. Considering the findings that the neointimal growth pattern in porcine coronary stents after 1 month was compatible with those in human coronary stents after 6–12 months [21], the results of the present serial study (1, 3, and 6 months follow-up after stent implantation) in porcine coronary arteries may be compatible with very late (beyond approximately 2 or 3 years follow-up) neointimal responses in DES-treated patients. In the present study, NIH thickness in the analyses of whole segments and NIH volume in the analyses of maximal NIH segments in EES-implanted lesions were maximal at 3 months and decreased at 6 months. To the best of our knowledge, this is the first report to show a biphasic neointimal response of EES-implanted segments, particularly neointimal regression between 3 and 6 months in the porcine coronary model. In addition, neointimal regression occurred with a morphological shift from a layered pattern to a homogeneous pattern. The mechanism of the neointimal regression is not clearly understood. Recent OCT study reported that the evolution of heterogeneous to homogeneous neointima was observed in lesions with NIH regression, while the evolution of homogeneous to layered neointima was detected in lesions with NIH progression (*P* < 0.001) [22]. The present study supports the notion that neointimal stabilization from immature tissue, such as fibrin near strut, rich extracellular matrix, and inflammatory cells to maturation with rich smooth muscle cells, may play a role in neointimal regression [23].

*Limitations*. This study is based on the nonatherosclerotic normolipemic porcine coronary model. Therefore, the results of the present study may need to be interpreted with caution for clinical practice of DES-treated patients. Although the diseased porcine animal models have been increasingly used for the evaluation of DES and provided more similar biological responses, normolipemic swine coronary artery is still recommended as the choice of the porcine model to evaluate tissue response in consensus documents [24, 25]. The comparisons between OCT images with histology were not performed at each stage of follow-up.

## 4. Conclusion

This serial OCT study found that EES had a biphasic pattern (early progression and late regression) of neointimal tissue that correlated with changes in neointimal morphology.

## Figures and Tables

**Table 1:** Quantitative coronary angiographic and optical coherence tomographic findings of the entire stent segment at 1, 3, and 6 months after stent implantation.

	Bare-metal stent (*n* = 15)			Everolimus-eluting stent (*n* = 15)		
	1 month	3 months	6 months	1 month	3 months	6 months
Quantitative coronary angiography analyses						
Proximal reference, mm	2.7 (2.6–3.1)	2.8 (2.6–3.2)	2.8 (2.5–3.2)	2.9 (2.7–3.2)	3.0 (2.6–3.1)	2.7 (2.4–3.1)
Distal reference, mm	2.6 (2.4–2.8)	2.6 (2.3–3.0)	2.5 (2.2–2.9)	2.6 (2.4–2.8)	2.5 (2.4–2.7)	2.5 (2.3–2.7)
Minimal lumen diameter, mm	2.2 (2.0–2.5)	2.2 (2.1–2.4)	2.2 (2.0–2.4)	2.4 (2.0–2.6)∗	1.7 (1.4–2.3)	1.8 (1.5–2.2)
Optical coherence tomography findings						
Neointimal thickness, *μ*m	190.4 (127.0–243.9)	182.2 (131.9–229.4)	169.8 (112.7–211.1)	120.6 (93.8–203.9)∗	276.5 (172.2–428.1)	269.6 (199.9–396.4)
Stent area, mm^2^	6.1 (5.2–6.8)	6.1 (5.4–7.0)	6.0 (5.2–6.8)	6.2 (5.3–6.9)	6.3 (5.4–7.0)	6.3 (5.0–6.9)
Lumen area, mm^2^	4.5 (3.8–5.6)	4.7 (4.0–5.4)	4.6 (3.8–5.3)	4.9 (4.1–5.8)∗	3.8 (3.0–4.9)	3.8 (3.2–4.5)
Neointimal area, mm^2^	1.6 (1.1–1.9)	1.4 (1.0–1.9)	1.4 (0.9–1.7)	1.3 (0.9–1.7)∗	2.5 (1.4–3.6)	2.4 (1.6–3.1)
Percentage of neointimal hyperplasia area, %	25.3 (19.2–29.4)	23.9 (17.3–32.0)	23.2 (13.1–29.9)	21.5 (12.9–28.1)∗	39.1 (24.1–53.7)	35.4 (31.7–45.0)

*P < 0.05, using Friedman tests.

**Table 2:** Optical coherence tomographic findings of the maximum neointima site at 1, 3, and 6 months after stent implantation.

	Bare-metal stent (*n* = 15)			Everolimus-eluting stent (*n* = 15)		
	1 month	3 months	6 months	1 month	3 months	6 months
Quantitative analyses						
Stent volume, mm^3^	24.5 (21.4–27.4)	24.3 (21.8–26.9)	23.3 (21.4–26.5)	25.5 (20.7–27.2)	25.5 (20.4–27.6)	24.7 (19.9–27.9)
Lumen volume, mm^3^	16.7 (14.1–20.2)	17.5 (15.7–20.6)	17.6 (14.4–20.8)	18.7 (16.5–21.4)	12.6 (11.5–17.6)	15.5 (13.3–18.3)
Neointima volume, mm^3^	7.3 (4.7–8.4)	6.9 (4.6–8.2)	6.4 (4.3–7.4)	4.8 (4.1–7.6)	9.8 (5.7–15.1)	8.6 (6.0–11.2)
Qualitative analyses						
Homogeneous, %	12 (80.0)	14 (93.3)∗	15 (100)	11 (73.3)	5 (33.3)	12 (80.0)
Layered, %	3 (20.0)	1 (6.7)∗	0 (0)	4 (26.7)	10 (66.7)	3 (20.0)

*P < 0.05, comparing bare-metal stent with everolimus-eluting stent.
