# Corrigendum to “Serial Changes of Neointimal Tissue after Everolimus-Eluting Stent Implantation in Porcine Coronary Artery: An Optical Coherence Tomography Analysis”

**DOI:** 10.1155/2019/8629375

**Published:** 2019-11-21

**Authors:** Hoyoun Won, Jung-Sun Kim, Dong-Ho Shin, Byeong-Keuk Kim, Young-Guk Ko, Donghoon Choi, Yangsoo Jang, Myeong-Ki Hong

**Affiliations:** ^1^Chung-Ang University Medical Center, 102 Heukseokro, Dongjak-gu, Seoul 156-755, Republic of Korea; ^2^Division of Cardiology, Department of Internal Medicine, Severance Cardiovascular Hospital, Yonsei University College of Medicine, Yonsei University Health System, 250 Seongsanno, Seodaemun-go, Seoul 120-752, Republic of Korea; ^3^Cardiovascular Institute, Yonsei University College of Medicine, 250 Seongsanno, Seodaemun-go, Seoul 120-752, Republic of Korea; ^4^Severance Biomedical Science Institute, Yonsei University College of Medicine, 250 Seongsanno, Seodaemun-go, Seoul 120-752, Republic of Korea

In the article titled “Serial Changes of Neointimal Tissue after Everolimus-Eluting Stent Implantation in Porcine Coronary Artery: An Optical Coherence Tomography Analysis” [1], a research grant from Yonsei University, College of Medicine (6-2011-00217), should be added. The Acknowledgments section should be corrected as follows.

“This study was supported by a grant from the Korea Healthcare Technology R&D Project, Ministry for Health, Welfare and Family Affairs, Republic of Korea (nos. A085012 and A102064), grants from the Korea Health 21 R&D Project, Ministry of Health and Welfare, Republic of Korea (no. A085136), and the Cardiovascular Research Center, Seoul, Republic of Korea, and a research grant from Yonsei University, College of Medicine (6-2011-00217).”
